# Modulation of Emotion Perception *via* Amygdala Stimulation in Humans

**DOI:** 10.3389/fnins.2021.795318

**Published:** 2022-02-09

**Authors:** Krzysztof A. Bujarski, Yinchen Song, Tiankang Xie, Zachary Leeds, Sophia I. Kolankiewicz, Gabriella H. Wozniak, Sean Guillory, Joshua P. Aronson, Luke Chang, Barbara C. Jobst

**Affiliations:** ^1^Department of Neurology, Dartmouth-Hitchcock Medical Center, Geisel School of Medicine at Dartmouth, Lebanon, NH, United States; ^2^Department of Psychological and Brain Sciences, Dartmouth College, Hanover, NH, United States; ^3^Department of Quantitative Biomedical Sciences, Dartmouth College, Lebanon, NH, United States; ^4^Department of Surgery, Dartmouth-Hitchcock Medical Center, Geisel School of Medicine at Dartmouth, Lebanon, NH, United States

**Keywords:** amygdala, brain stimulation, affective valence, emotion perception, arousal

## Abstract

**Background:**

Multiple lines of evidence show that the human amygdala is part of a neural network important for perception of emotion from environmental stimuli, including for processing of intrinsic attractiveness/“goodness” or averseness/“badness,” i.e., affective valence.

**Objective/Hypothesis:**

With this in mind, we investigated the effect of electrical brain stimulation of the human amygdala on perception of affective valence of images taken from the International Affective Picture Set (IAPS).

**Methods:**

Using intracranial electrodes in patients with epilepsy, we first obtained event-related potentials (ERPs) in eight patients as they viewed IAPS images of varying affective valence. Next, in a further cohort of 10 patients (five female and five male), we measured the effect of 50 Hz electrical stimulation of the left amygdala on perception of affective valence from IAPS images.

**Results:**

We recorded distinct ERPs from the left amygdala and found significant differences in the responses between positively and negatively valenced stimuli (*p* = 0.002), and between neutral and negatively valenced stimuli (*p* = 0.017) 300–500 ms after stimulus onset. Next, we found that amygdala stimulation did not significantly affect how patients perceived valence for neutral images (*p* = 0.58), whereas stimulation induced patients to report both positively (*p* = 0.05) and negatively (< 0.01) valenced images as more neutral.

**Conclusion:**

These results render further evidence that the left amygdala participates in a neural network for perception of emotion from environmental stimuli. These findings support the idea that electrical stimulation disrupts this network and leads to partial disruption of perception of emotion. Harnessing this effect may have clinical implications in treatment of certain neuropsychiatric disorders using deep brain stimulation (DBS) and neuromodulation.

## Introduction

One of the most extensively studied regions in the human brain with respect to anatomy and function is the amygdala (McDonald, 1998; Murray, 2007; Benarroch, 2015; Janak and Tye, 2015). Extensive evidence from non-human and human animal studies implicates the amygdala in a wide variety of cognitive functions, including for identifying emotional information from the environment (LaBar et al., 1998; Tye and Janak, 2007; Fusar-Poli et al., 2009; Salzman and Fusi, 2010). Perception of emotion from environmental stimuli can occur in any sensory modality including visual, auditory, and olfactory and includes variety of components such as perception of affective valence, arousal, or dominance (Plutchik, 2001). The perception of affective valence, or just valence, refers to the intrinsic attractiveness/“goodness” (positive valence) or averseness/“badness” (negative valence) of events, objects, or situations (Frijda, 1986). The importance of the amygdala in this process is evidenced by studies in non-human primates demonstrating single neuron activations in the basolateral nucleus of the amygdala responding to the valence of environmental stimuli (Paton et al., 2006; Shabel and Janak, 2009). Likewise, functional MR studies in normal human subjects show amygdala activation during valence judgments (Canli et al., 2000; Garavan et al., 2001; Hariri et al., 2002; Phelps and LeDoux, 2005; Sergerie et al., 2008; Ball et al., 2009; Salzman and Fusi, 2010; Murray et al., 2014). Lastly, studies describing human subjects with acquired destructive amygdala lesions demonstrate deficits in perception of negatively valenced images—such as faces—with subjects reporting them as less fearful or less threatening (Adolphs et al., 1994; Hamann et al., 1999; Canli et al., 2000; Anderson and Phelps, 2001; Kilpatrick and Cahill, 2003; Kensinger and Corkin, 2004; Phelps, 2004; Richardson et al., 2004; LaBar and Cabeza, 2006; Sergerie et al., 2006).

An important source of evidence for the role of the amygdala in human cognition has come from studies using intracranial EEG. EEG recordings using electrodes inserted directly into the human amygdala have provided evidence of activation to both negatively and positively valenced images (Halgren et al., 1978; Oya et al., 2002; Krolak-Salmon et al., 2004; Pourtois et al., 2010; Sato et al., 2011; Guillory and Bujarski, 2014). In addition, studies of electrical stimulation delivered using intracranial EEG electrodes to the human amygdala have offered additional insights into the organization of valence. For instance, Lanteaume et al. (2007) used a validated scale to measure the effect of 50 Hz electrical stimulation of the right and left amygdala. The investigators reported that emotional states such as fear, anxiety, sadness, and joy can be induced by amygdala stimulation (Lanteaume et al., 2007). Similar results have been reported by other investigators (Oya et al., 2002; Meletti et al., 2005; Naccache et al., 2005).

Despite abundant evidence that electrical stimulation of the human amygdala can induce in some instances internal emotional states, the effect of amygdala stimulation on *perception of emotion from external environmental stimuli* has not been well investigated. In a single patient case report, Bijanki et al. (2014) studied a patient with intracranial electrodes located in the right amygdala and found that stimulation enhanced positive valence during viewing of faces, i.e., the subject perceived faces more positively when stimulation was applied. This effect of stimulation on perception of valence was measured in the absence of induction of any internal emotional states (Bijanki et al., 2014).

Building upon work cited above, the objective of this study was to investigate how electrical stimulation of the human amygdala effects perception of emotion from the environment. To accomplish this objective we first measured evoked responses (ERPs) from three regions known to be important in processing emotion (left amygdala, the left hippocampus and the left ventromedial prefrontal cortex) during viewing of images taken from the International Affective Picture Set (IAPS). Next, in a separate group of patients, we applied electrical stimulation to the left amygdala to determine its effect on perception of valence from same IAPS images. We chose to focus the study on the left amygdala as prior evidence suggests possible distinct roles in valence perception between the right and the left amygdala (Meletti et al., 2006). Our initial working hypothesis—as gained from previous studies discussed above—was that stimulation of the amygdala would augment the reported valence of IAPS images leading to more negative or more positive valence judgments.

## Materials and Methods

The study recruited 18 consecutive patients at Dartmouth-Hitchcock Medical Center undergoing intracranial EEG evaluation for epilepsy surgery who fit the inclusion criteria. Inclusion criteria included both female and male patients who were willing to participate, able to consent, had electrodes localized to the left amygdala, and had pre-surgical FSIQ greater than 70.

Electrodes used for the study were either Ad-Tech SEEG electrodes 0.86 mm diameter with 10 contacts per electrode or PMT SEEG electrodes 0.8 mm diameter 8–12 contacts per electrode.

**FIGURE 1:**
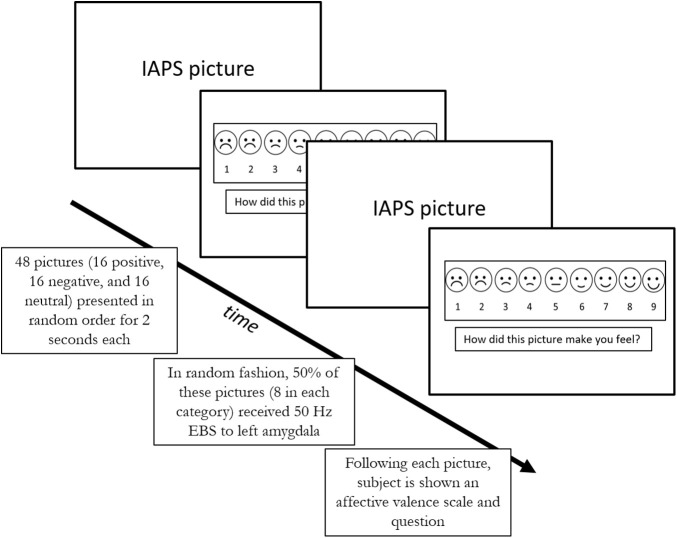
The Affective Valence Perception Task.

The task was designed *de novo* for the purpose of this study (Figure 1). 48 images were selected from the International Affective Picture System (IAPS) (Bradley and Lang, 2007) using the following criteria: images of scenes with people only, normal control affective valence values between 2 and 8 (1 and 9 were excluded due to extreme nature of some images). Due to the fragile nature of our patients, we also excluded images with the following criteria: stimuli with medical themes (i.e., hospital themes), stimuli with animals, sexuality, and torture. The following images were selected from the IAPS picture set: 2102, 2190, 2038, 2191, 2235, 2272, 2305, 2357, 2381, 2396, 2485, 2635, 2513, 8232, 2435, 1340, 2040, 2070, 2091, 2150, 2208, 8370, 8350, 8030, 2299, 2224, 8490, 8120, 7325, 2170, 2391, 2095, 2141, 2455, 2683, 3180, 3500, 3530, 6212, 6312, 9050, 6831, 9428, 9800, 9900, 9520, 6838. The images were divided into three categories based on their specific IAPS valence rating: neutral used IAPS value 4.1–6.0, positive 6.1–8.0, and negative 2.0–4.0. The entire picture set was approved by the DHMC IRB. Prior to presentation, each patient was shown sample images and could abort the study at any time during the task. Average IAPS values for the final picture set for neutral category was 5.23 (range 4.50 to 5.84), for positive category was 7.11 (range 6.31 to 7.69) and for negative category was 2.37 (range 1.78 to 2.98).

All tasks were administered between June 2015 and April 2018 at Dartmouth-Hitchcock Medical Center in the Epilepsy Monitoring Unit during an approximately 10-day admission for epilepsy surgery evaluation. Following informed consent, a time was chosen when the subject was able to participate in the study. Generally, this occurred when antiepileptic drugs were in the process of being weaned and the patient was awaiting the occurrence of seizures.

A laptop computer running SuperLab 5 (Cedrus Corporation, 2015) was connected to a 19.5 inch display monitor which was viewed by the patient at a distance of approximately three feet. The subject was given a separate numerical key pad for providing responses. The laptop computer was connected to StimTracker which provided a TTL pulse for triggering the Grass S12X stimulator (FDA approved for clinical use) and separately placing stimulus markers on the EEG. Neuroworks software was used for EEG recording and for electrode selection for stimulation.

Coregistration of CT to MRI was used to determine optimal electrode contacts for stimulation. Pre-operative whole-brain T1-weighted MRI scans were carried out on a 3-T scanner (Siemens Prisma) using a 3D BRAVO sequence with TR = 8.4 ms, TE = 3.4 ms, voxel size = 0.5 × 0.5 × 1.25 mm^3^. Spine echo T2-weighted coronal MRI scans were also performed to focus on the amygdala and hippocampus areas with TR = 4,582 ms, TE = 99 ms, and voxel size of 0.35 mm × 0.35 mm × 1.5 mm. All scans were visually inspected by board-certified radiologists for abnormalities, motion and artifacts. There was no evidence that motion or other artifacts existed in the scans from these ten patients. The left amygdala sub-field segmentation was conducted on the T1-weighted and T2-weighted scans using the Freesurfer tool (Saygin et al., 2017). Post-operative CT head scans were obtained with a voxel size of 0.5 × 0.5 × 0.5 mm^3^ to show locations of depth electrodes. Pre-operative T1-weighted whole-brain MRI scans and post-operative CT head scans were co-registered using SPM12 toolbox in Matlab 2017b. The locations of the depth electrodes were determined by thresholding the registered CT images. Each depth electrode in the registered CT images was represented as a cluster of scattered dots. The coordinates of each depth electrode were calculated by averaging the coordinates of all the dots within each cluster. Individual T1-weighted MRI scans were normalized to Montreal Neurological Institute (MNI) space in SPM12. For visualization purposes, depth electrode coordinates were also transformed to MNI space.

### Specific Methods for Study 1—ERP Analysis

For Study 1, the task was presented to an initial cohort of eight patients without electrical stimulation to obtain ERPs. The image set was presented using SuperLab randomization settings (i.e., by randomization of presented trials in each block). EEG data was exported in EDF format for analysis. Behavioral data was exported in Excel format for analysis.

EEG data was initially recorded using a Natus EEG system sampled at 2 KHz and down-sampled to 512 Hz to speed up computations. Channels were visually examined and bad channels with excessive noise or epileptic activities were removed. Next, we used a finite impulse response (FIR) filter to remove power line noise at 60 Hz, 120 Hz and 180 Hz. We applied global average re-reference to the filtered data. We selected channels located within the left amygdala, hippocampus, and ventromedial prefrontal cortex (vmPFC), resulting in 39 electrodes in amygdala, 81 electrodes in hippocampus and 49 electrodes in vmPFC. Segmentation was performed based on stimulus markers appropriate to each valence category. Time windows of 500 ms before and 1,000 ms after the onset of stimulus marker were segmented. For each channel, epochs with amplitude values greater than ± 50 μV were rejected. Data was baseline corrected with a time interval of 250 ms spanning from 500 to 250 ms prior to the stimulus onset. Epochs were averaged across valence categories for each channel.

Across three different anatomical regions (hippocampus, left amygdala and prefrontal), we compared the ERP responses between each pair of valence categories across eight subjects. We assessed if there were any time intervals with significant differences with permutation cluster test. Permutation cluster test is a non-parametric statistical test that identifies continuous time points which are significantly different between conditions, without causing the multiple comparison problems (Maris and Oostenveld, 2007). For each time point a repeated 1-sample *t* test was performed between the two conditions, and continuous time points whose *t*-values above a threshold *t* value corresponding to an uncorrected *p* value of 0.05 were grouped into clusters. Each cluster consists of at least two adjacent time points. The *t* values for each cluster are then summed to become a cluster-level statistic.

To approximate the significance of cluster level statistic, we randomly shuffled the condition labels of the ERP time points and recorded the largest possible cluster-level statistics for each permutation. We repeated the procedure 5,000 times and formed permutation distribution with the largest possible cluster-level statistics. We then calculated the *p* values for clusters from the original dataset based on the permutation distribution.

All analyses were performed in Python environment v3.7 using the MNE package (Gramfort et al., 2013).

### Specific Methods for Study 2—Amygdala Stimulation

The task was presented to a second cohort of 10 patients with electrical stimulation of the left amygdala. As stimulation causes artifact, EEG data from the stimulation trials could not be analyzed. For all 10 patients, in a random order, 50% of the encoding images received electrical stimulation (i.e., by random assignment of “stimulate” or “not stimulate” to each trial). Stimulation of the amygdala was time-locked to the presentation of each image for duration of 2 s matching the time of image presentation. Time between image presentations in the encoding task was 10 s (i.e., the inter-trial interval).

The choice of electrode pair used for recording and stimulation was made based on their positions in the co-registered images. Generally, this included electrodes which were most centered in the left amygdala. No systemic attempt was made to select contacts is a specific sub-nucleus of the amygdala given inherent error of co-registration and normalization.

Optimal stimulation parameters were defined by intensity which were safe for clinical applications, did not induce electrographic seizures (afterdischarges) or clinical seizures and did not produce subjective symptoms by patient self-report. A choice of 50 Hz stimulation frequency, 0.3 ms pulse duration, and alternating pulse morphology was made for all patients in this study as these parameters are routinely utilized for stimulation mapping at Dartmouth-Hitchcock and are known to be safe for clinical applications. Due to limitations of the available Grass 12X stimulator, stimulation frequencies commonly used for DBS (120–180 Hz) were not used. Based on stimulation safety data provided by both electrode manufacturers, we limited maximum stimulation intensity to 5 mA. This stimulation intensity of 5 mA is in the range commonly used for clinical purposes and is well under the 40 microcoulombs per square centimeter stimulation intensity known to possibly cause injury to tissue.

The initial portion of the experiment involved identifying the optimal stimulation intensity for each patient. Starting at 1.0 mA, the chosen electrodes were stimulated in bipolar fashion for 2 s observing for electrographic seizures, clinical seizures, and patient report of any subjective symptoms. Duration of 2 s was used because this was the duration of image presentation in the task. If no electrographic seizures, symptoms, or clinical seizures occurred, stimulation intensity was increased stepwise by 1 mA up until either afterdischarges were seen, subjective symptoms were reported by the patient, or the maximum of 5 mA stimulation intensity was reached. Stimulation intensity was chosen for each patient individually based on 80% of after-discharge threshold or maximum of 5 mA.

Response data was exported from SuperLab to Excel for analysis. Affective valence judgments for each image were separated into two categories: stimulated and unstimulated. For each patient, stimulation was randomly assigned for each image; therefore, the stimulated image for patient 1 were different from those for patient 2, and so on. Images were grouped into valence categories as per original image selection (see image selection in Methods above). With a linear mixed effects model, we quantitatively assessed how valence judgment score for each picture change with stimulation status and the picture’s valence category and possible interactions between the two. In the mixed effects model, the valence category and stimulation status are treated as fixed factors and subject is treated as random factor and modeled with random intercepts. We further calculated pairwise difference in means between terms and its associated confidence intervals with *post hoc* comparison tests (in our case, with least-squares means comparisons). The statistical analyses were conducted in R language (R Core Team, 2014) with lme4 (Bates et al., 2015) and lmerTest (Kuznetsova et al., 2017) packages. We conducted model assumption tests for our mixed effects model by checking 1. The homogeneity of variance and 2. Normality of residuals. We do not find any violations.

## Results

Table 1 shows the demographics of 18 patients who met inclusion criteria and were enrolled into Study 1 and Study 2. Patients included men and women of differing epilepsy diagnosis with IQ ranging from 77 to 112 and a variety of seizure onset zones including temporal lobe epilepsy, bilateral temporal lobe epilepsy, occipital lobe epilepsy, and frontal lobe epilepsy.

**TABLE 1:** Demographics and afterdischarge threshold testing.

Subject	Age	Gender	Epilepsy type	FSIQ	Afterdischar ge threshold (mA)	Stimulation induced seizures	Final stimulation intensity (mA)	Final stimulation intensity (uC/cm^2^)	Symptoms elicited by final stimulation
**Part 1**									
1	53	M	L TLE	112	–	–	–	–	–
2	22	M	R TLE	101	–	–	–	–	–
3	56	F	L FLE	105	–	–	–	–	–
4	37	F	L FLE	97	–	–	–	–	–
5	32	M	L TLE	89	–	–	–	–	–
6	33	F	Bitemporal	102	–	–	–	–	–
7	23	M	L TLE	81	–	–	–	–	–
8	38	F	L TLE	77	–	–	–	–	–
**Part 2**									
1	50	M	L TLE	84	4	Yes	3	17.9	none
2	41	F	R TLE	72	4	No	3	17.9	none
3	22	F	R TLE	99	2	No	1	5.9	none
4	45	M	L TLE	100	4	No	3	17.9	none
5	37	M	L FLE	96	4	No	3	17.9	none
6	29	M	L FLE	102	5	No	5	29.8	none
7	49	M	L FLE	107	2	Yes	1	5.9	none
8	41	F	L TLE	97	2	No	1	5.9	none
9	27	F	L OLE	100	4	No	1	5.9	none
10	49	F	R TLE	98	3	No	2	11.9	none

*TLE is temporal lobe epilepsy, FLE is frontal lobe epilepsy, OLE is occipital lobe epilepsy, mA is milliamps, uC/cm^2^ is microcoulombs per square centimeter.*

### Study 1—ERP Analysis

**FIGURE 2:**
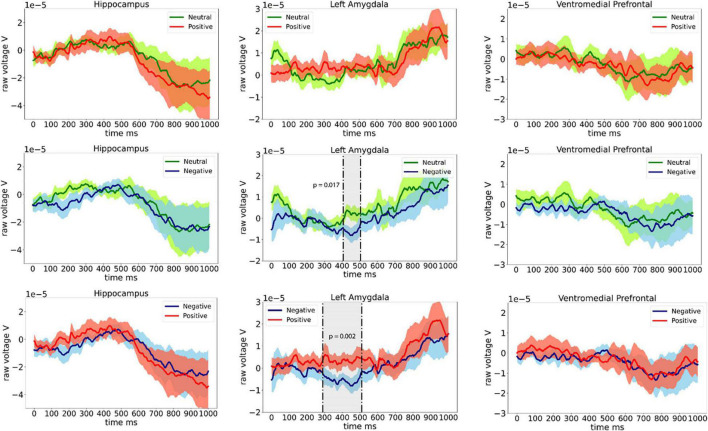
Event related potentials (ERPs) recorded from the left hippocampus, left amygdala, and the left ventromedial prefrontal cortex from eight patients.

Grand average ERPs in eight patients from the left amygdala, left hippocampus, and left ventrolateral prefrontal electrodes are shown in Figure 2. Significant difference in ERP between Neutral and Negative valence images (permutation cluster test, *p* = 0.017) was observed approximately 400–500 ms following stimulus onset. Furthermore, significant difference between Negative and Positive valence images (permutation cluster test, *p* = 0.002) was observed approximately 300–500 ms following stimulus onset in the left amygdala. No differences were observed between Neutral and Positive stimuli in the left amygdala (permutation cluster test, *p* = 0.853). Also, no differences in ERPs were observed in the left hippocampus and the left ventromedial prefrontal cortex.

### Study 2—Amygdala Stimulation

Final stimulation intensity for each of the 10 patients is shown in Table 1. In 9 of 10 patients, brief self-limited electrographic seizures or “afterdischarges” were eventually seen with stimulation and the amplitude was reduced to approximately 80% of the afterdischarge threshold. Such afterdischarges are common occurrences during functional mapping procedures for clinical applications. The average afterdischarge threshold was 3.4 mA (about 17 microcoulombs per square centimeter of brain tissue) and ranged between 2–5 mA (range of 11.9–29.8 microcoulombs per square centimeter of brain tissue). Two patients experienced focal seizures during this part of the experiment (patient 1 and patient 7). Both times, the semiology or clinical manifestations of the seizures matched the patient’s natural seizure semiology and were obtained ipsilateral to the patient’s own seizures focus. There were no adverse effects of the seizures. Similar to afterdischarges, seizures are common during stimulation-based functional mapping for clinical applications. The study was successfully completed in both patients who experienced seizures approximately 1 day later at lower stimulation intensities. Final stimulation parameters did not produce any electrographic seizures or self-reported subjective symptoms.

**FIGURE 3:**
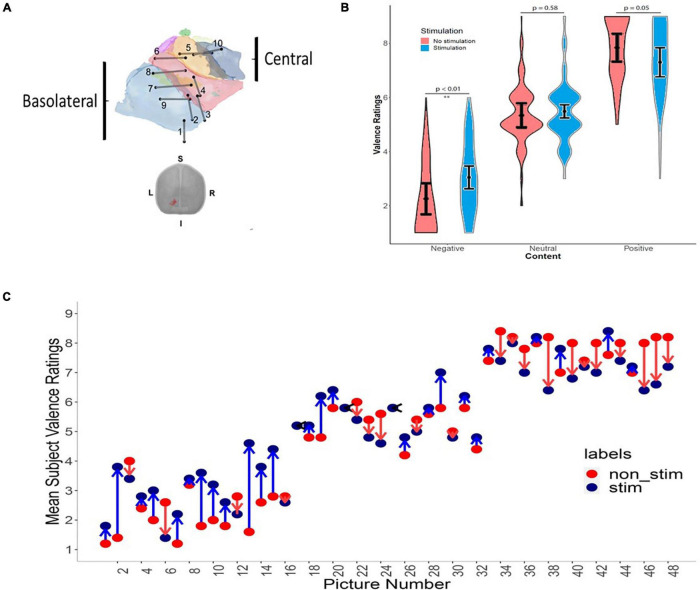
The effect of amygdala stimulation on perception of valence. **(A)** The location of left amygdala electrode contacts for 10 patients. **(B)** Violin plot showing average valence rating across 10 patients for all 48 images separated by valence categories and stimulation status (neutral used IAPS value 4.1–6.0, positive 6.1–8.0, and negative 2.0–4.0). **(C)** Mean subject valence ratings across all patients for all images for unstimulated and stimulated trials. X-axis represents images 1–48 arranged in order of their original IAPS valence score from negative on the left to positive on the right. The y-axis shows mean patient-reported valence judgments ranging from one (negative) to nine (positive) for both non-stimulated (red) and stimulated (blue) trials. The arrows represent the average direction and magnitude of change between the unstimulated and stimulated trials. Blue arrow represents an increase in mean valence (i.e., more positive) with stimulation, red arrow represents a decrease in mean valence (more negative) with stimulation, and black arrow represents no change in mean valence with stimulation.

Electrode location and effects of stimulation on reporting of valence are shown in Figure 3. For most patients, both stimulating electrode pairs were within the amygdala, although for patients 1, 2, 4 one of the electrodes was within the amygdala, while the second was outside of the structure (Figure 3A). In these patients, the size of the electrical field generated by stimulation was likely smaller and akin to mono-polar stimulation. These patients were included in the analysis as volume of tissue activated (VTA) models in deep brain stimulation predict an electrical field around each one of the two electrodes in a stimulation pair (Butson and McIntyre, 2008). Figure 3B shows a violin plot of average valence judgments across all 10 patients for three different valence categories for stimulated and unstimulated images. Figure 3C shows the average reported valence for each image numbered 1–48 averaged across all 10 patients separated by stimulation status. Table 2 shows the results of the *post hoc* tests of stimulation on perception of valence and delayed recognition. Stimulation did not have any significant effect on judgment of valence intensity for images in the neutral category (mean difference between non-stimulated and stimulated group was 0.15, *p* = 0.58). In the positive category, stimulation induced an average decline in reported valence toward neutral (Tukey’s *post hoc* comparison test, mean difference between non-stimulated and stimulated group = −0.54, *p* = 0.05). In the negative valence category, stimulation induced a significant increase in reported valence toward neutral (Tukey’s *post hoc* comparison test, mean difference between non-stimulated and stimulated group = 0.79, *p* = 0.01.

**TABLE 2:** The effect of stimulation of the left amygdala on valence judgments for negative, neutral, and positive images.

	Estimate	Lower 95% CI	Upper 95% CI	*p* value	Degree of freedom
Negative valence	0.79	0.24	1.33	< 0.01	45
Neutral valence	0.15	–0.40	0.70	0.58	45
Positive valence	–0.54	–1.08	< 0.01	0.05	45

## Discussion

We used intracranial EEG in patients undergoing assessment for epilepsy surgery to study one aspect of perception of emotion from visual images—affective valence—with a goal to understand how electrical stimulation of the amygdala effects such perception. Consistent with prior literature, differences in event-related potentials (ERPs) between Negative and Neutral and Negative and Positive images recorded from the left amygdala support the idea that these brain regions are part of a neural network specialized for perception of valence, especially negative valence (Krolak-Salmon et al., 2004; Pourtois et al., 2010; Jung et al., 2011). Contrary to our working hypothesis, we found that low-intensity 50 Hz electrical stimulation of the amygdala *diminished* the intensity of perceived valence or environmental stimuli, in effect rendering items from the environment as more neutral.

### Disruption of Amygdala Function by Stimulation

The effect of electrical stimulation of neural structures on behavior likely relates to the underlying neural organization and the exact parameters of stimulation. For instance, it is well recognized that 50 Hz stimulation of the primary hand area of the human motor cortex results in contralateral hand dystonia, i.e., clinical activation. In contrast, similar 50 Hz stimulation over Broca’s area generally leads to interruption of language function, i.e., clinical inhibition (Havas et al., 2015). The structure of the amygdala, i.e., compact grouping of functionally distinct neurons with either corticoid or nuclear histology amends well to disruption of function by electrical stimulation. This is consistent with our main finding, i.e., that subthreshold (i.e., below afterdischarge threshold and below production of subjective symptoms) 50 Hz unilateral stimulation of the left amygdala changed how patients reported the valence of images. In essence, stimulation caused patients to report both positively and negatively valenced stimuli as more neutral. The effect observed is contrary to our initial working hypothesis that electrical stimulation would augment the perceived valence of the IAPS images. The effect is similar to previously described effects of destructive amygdala lesions on perception of valence and are likely related to stimulation-induced disruption of amygdala function and the downstream consequences on neural networks important for perception of valence (Adolphs et al., 1997; LaBar and Phelps, 1998; Phelps et al., 1998; Anderson and Phelps, 2001). This effect is similar to hippocampal stimulation studies in human subjects which have shown that stimulation causes disruption of episodic memory (Halgren and Wilson, 1985; Jacobs et al., 2016). In addition, the effect is similar to that seen in deep brain stimulation (DBS) studies of subcortical structures in Parkinson disease where effect of high frequency stimulation mimic a lesion (Dostrovsky and Lozano, 2002; Herrington T. M. et al., 2016).

### Comparison to Prior Studies

The behavioral effects on valence perception obtained by amygdala stimulation in this study are distinct from several prior human intracranial EEG studies. Bijanki et al. (2014) reported right amygdala stimulation induced augmentation of perceived positive valence from images (Bijanki et al., 2014). This finding is contrary to our results and may be related to lateralized specialization of amygdala function. Prior intracranial EEG studies provide evidence of cerebral hemispheric functional lateralization for processing valence (Guillory and Bujarski, 2014). For instance, Lanteaume et al., reported that increasing the intensity of electrical stimulation of the right amygdala eventually induced subjective experiences of uniquely negatively valenced emotional states while left amygdala stimulation induced either positively or negatively valenced emotional states (Lanteaume et al., 2006). Such lateralization of amygdala function is supported by other intracranial EEG studies (Oya et al., 2002; Naccache et al., 2005; Meletti et al., 2006). As such, the distinct effects of stimulation of right and left amygdala stimulation may be related to distinct roles of the right and left amygdala in processing of positive and negative valence and how stimulation disrupts these processes.

In an important study measuring the physiological and behavioral effects of electrical stimulation of the human amygdala on memory, Inman et al. (2018) reported that stimulation of the amygdala during perception of neutrally valenced images enhanced delayed memory (Inman et al., 2018). The investigators postulated that stimulation induced interactions between the amygdala, hippocampus, and entorhinal cortex which in turn resulted in memory enhancement. In the study, Inman et al. (2018) used burst-stimulation composed of eight per second bursts of four pulses at 50 Hz. Compared to continuous stimulation used in the present study, burst stimulation has been demonstrated to induce hippocampal LTP in rat models (Larson et al., 1986) and replicates typical characteristics of hippocampal physiology, i.e., the theta arousal rhythm (Green and Arduini, 1954). Therefore, differences in stimulation parameters by Inman et al. (2018) and the current study may be responsible for the distinct effects of amygdala stimulation, i.e., augmentation of versus disruption of amygdala function.

### Implications to Treatment of Psychiatric Disorders

Despite advances in pharmaceutical options and novel forms of psychotherapy, a large proportion of patients with psychiatric conditions do not adequately respond to standard treatment (Al-Harbi, 2012). For persons who have not benefited from conventional intervention, DBS is an emerging treatment option (Altinay et al., 2015; Cleary et al., 2015; Coenen et al., 2015; Graat et al., 2017). Despite the successful use of DBS in many neurological disorders, recent clinical trials in psychiatry have shown limited benefits (Graat et al., 2017; Widge et al., 2018). The main obstacle limiting the use of DBS in psychiatry is poor understanding of the underlying anatomy of neural networks involved in psychiatric disorders. Intracranial EEG in patients undergoing an evaluation for epilepsy surgery as used in this study may help to further advance our understanding targeting for DBS.

Mounting evidence from functional imaging studies shows that dysfunctional processing of affective valence may be a common mechanism for a vast array of psychiatric illnesses. Functional imaging studies in patients with anxiety disorders have shown increased amygdala BOLD signal activation to negatively valenced stimuli as compared to controls (Bishop et al., 2005; Stein et al., 2007), lack of amygdala activation to positively valenced stimuli with higher-state anxiety levels (Somerville et al., 2004), and abnormal amygdala activation in patients with anxiety to neutral stimuli (Cooney et al., 2006). In patients with autistic spectrum disorder, symptoms of social anxiety highly correlate with abnormal amygdala activation to positively and negatively valenced social stimuli (Herrington T. M. et al., 2016, 2017a,b). Multiple studies in patients with post-traumatic stress disorder have found correlations between increased activation of the amygdala to negatively valenced stimuli with increasing disease severity, and decrease in activation with improvement of symptoms (Pissiota et al., 2002; Protopopescu et al., 2005; Shin et al., 2005; Francati et al., 2007; Langevin et al., 2016). Lastly, studies in persons with major depressive disorder show that the amygdala, along with the subcallosal cingulate, may be important in the production of negatively valenced emotions (Davey et al., 2015).

Two groups have reported results of long-term amygdala stimulation for treatment of psychiatric disorders. Strum et al., reported the outcome of bilateral amygdala DBS in a non-verbal autistic 13-year-old patient with severe self-injurious behavior (Sturm et al., 2012). Multiple sub-nuclei or the right and left amygdala were continuously stimulated using 130 Hz frequency and 120 μs duration up to 6.5 volts amplitude. Bilateral stimulation of the basolateral nuclei was found to be most successful in improving self-injurious behavior and other psychiatric symptoms of autism over a period of 24 months of follow-up. Langevin et al., described a patient with severe refractory PTSD who underwent bilateral stimulation of the basolateral nucleus of the amygdala using frequency of 160 Hz and pulse width of 60 μs with stimulation amplitude of 1.4 V (Langevin et al., 2016). At 8 months of follow-up, the patient experienced significant improvement in the Clinician-Administered PTSD Scale.

The results from the current study support further investigation of amygdala DBS for treatment of specific intractable neuropsychiatric conditions. As discussed above, abnormal perception of valence—especially negative valence—may be a common feature of anxiety-spectrum disorders such as PTSD. Our results suggest that electrical stimulation of the amygdala may disrupt such abnormal perception of negative valence an in turn modulate downstream neural networks important for production of disabling symptoms in this condition.

### Limitations

The first major limitation to this study is inherent to electrical brain stimulation itself. The exact extent of current flow from the electrodes through the brain and how such currents interact with cerebral architecture is not known. We are uncertain if stimulation exerted effects on the amygdala alone, a portion of the amygdala, or on neighboring structures such as the hippocampus, the entorhinal cortex, or even somewhat distant cerebral networks such as the ventromedial prefrontal lobe *via* connected white matter pathways. Future studies should employ volume of tissue of activation modeling to more precisely understand how stimulation parameters and specific location within the amygdala change the behavioral effect (Butson and McIntyre, 2008).

A second major limitation of this study is that we do not know if the effect of amygdala stimulation observed are truly related to perception of affective valence or if they are related to the effects of stimulation on another cognitive domain, such as arousal. Arousal refers to the psychological state of wakeful attention and is important for detection of salience, or the relative importance and novelty of stimuli (LaBar and Cabeza, 2006; Uddin, 2017). Studies show that the arousal/salience circuit includes the amygdala, insula, cingulate, and basal forebrain (Cuthbert and Insel, 2013; Uddin, 2017). Information related to the salience of the stimuli, i.e., arousal, was not captured in this study. Therefore, it is possible that the effect of stimulation on perception of both positively and negatively valenced stimuli seen in our study may be explained by stimulation induced disruption of arousal. Future studies on the effect of electrical stimulation of the amygdala will need to measure a wider range of cognitive domains.

A third major limitation of this study is the stimulation parameters used which were limited by the Grass 12X stimulator. Although FDA approved for clinical use, the stimulator delivers a maximum stimulation frequency of 50 Hz. In the present study, 8 of 10 patients experienced afterdischarges and two patients experienced seizures during stimulation (see Table 1). In fact, studies show that this stimulation frequency is prone to generation of seizures (Mohan et al., 2020). Higher stimulation frequencies between 100–200 Hz are likely produce a similar clinical effect with lower chance of seizure induction (Sun and Morrell, 2014). For this reason, commercial Medtronic™ DBS systems and Neuropace™ RNS systems use frequencies at between 130–200 Hz (Sun and Morrell, 2014). Future studies should use stimulation parameters which are likely to produce similar clinical effects yet are less likely to induce seizures.

A fourth major limitation is a general concern related to whether the amygdala should be considered a good target for DBS due to the many possible adverse effects of stimulation in this region. Evidence from many studies of amygdala stimulation in humans clearly demonstrate that a range of negative emotional experiences may occur with amygdala stimulation (Oya et al., 2002; Meletti et al., 2005; Naccache et al., 2005). Furthermore, investigators have recently reported cessation of breathing, i.e., central apnea, during stimulation of the central nucleus of the amygdala raising concerns that this brain region may be important in sudden unexpected death in epilepsy (Dlouhy et al., 2015; Nobis et al., 2018). In order to mitigate these potentially severe complication of amygdala stimulation, future studies should aim to target electrode placement and stimulation parameters to affect specific sub-nuclei of the amygdala such as the lateral nucleus, while avoiding stimulation of the central nucleus which is likely responsible for major side effects of electrical stimulation in this brain region.

A fifth major limitation of this study refers to the generalizability of these findings to treatment of any specific cognitive or psychiatric disorder. Although our results offer new evidence that electrical stimulation of the amygdala affects perception of valence, we do not understand if this effect would in any way benefit patients with specific cognitive or psychiatric conditions. More extensive studies and clinical trials of amygdala DBS targeting specific conditions need to be conducted.

### Future Directions

Rapid development of novel devices and surgical techniques are ushering in an era of targeted neuromodulation in cognitive neurology and psychiatry. Intracranial EEG in patients with epilepsy offers an opportunity to assess prospective targets for clinical trials employing DBS technology that can then be applied to patients suffering from intractable cognitive and psychiatric disorders. Future trials of amygdala stimulation should target specific sub-nuclei of the amygdala for neuromodulation, should use stimulation parameters which do not cause long term epileptogenic side effects, and should address safety concerns inherent to amygdala stimulation.

## Data Availability Statement

The raw data supporting the conclusions of this article will be made available by the authors, without undue reservation.

## Ethics Statement

The studies involving human participants were reviewed and approved by Dartmouth-Hitchcock Institutional Review Board. The patients/participants provided their written informed consent to participate in this study.

## Author Contributions

KB developed the idea and carried it out and wrote the manuscript. YS provided the images for Figures 2, 3. TX provided the statistical analysis. ZL developed the task and contributed in the programing. SK and GW developed the task and analyzed the data. SG developed the experimental set up. JA implanted the electrodes. LC assisted in overall study design and analysis. BJ reviewed the overall study. All authors contributed to the article and approved the submitted version.

## Conflict of Interest

The authors declare that the research was conducted in the absence of any commercial or financial relationships that could be construed as a potential conflict of interest.

## Publisher’s Note

All claims expressed in this article are solely those of the authors and do not necessarily represent those of their affiliated organizations, or those of the publisher, the editors and the reviewers. Any product that may be evaluated in this article, or claim that may be made by its manufacturer, is not guaranteed or endorsed by the publisher.
